# A Cost Reduced Variant of Epi-Genotyping by Sequencing for Studying DNA Methylation in Non-model Organisms

**DOI:** 10.3389/fpls.2020.00694

**Published:** 2020-05-28

**Authors:** Olaf Werner, Ángela S. Prudencio, Elena de la Cruz-Martínez, Marta Nieto-Lugilde, Pedro Martínez-Gómez, Rosa M. Ros

**Affiliations:** ^1^Laboratory of Molecular Systematics, Phylogeography and Conservation in Bryophytes, Department of Plant Biology, Faculty of Biology, University of Murcia, Murcia, Spain; ^2^Laboratory of Fruit Tree Breeding, Department of Plant Breeding, CEBAS-CSIC, Murcia, Spain

**Keywords:** DNA methylation, epi genotyping by sequencing, population genetics, reduced representation bisulfite sequencing, non-model organisms, *Prunus dulcis*

## Abstract

Reference-free reduced representation bisulfite sequencing uses enzymatic digestion for reducing genome complexity and allows detection of markers to study DNA methylation of a high number of individuals in natural populations of non-model organisms. Current methods like epiGBS enquire the use of a higher number of methylated DNA oligos with a significant cost (especially for small labs and first pilot studies). In this paper, we present a modification of this epiGBS protocol that requires the use of only one hemimethylated P2 (common) adapter, which is combined with unmethylated barcoded adapters. The unmethylated cytosines of one chain of the barcoded adapter are replaced by methylated cytosines using nick translation with methylated cytosines in dNTP solution. The basic version of our technique uses only one restriction enzyme, and as a result, genomic fragments are integrated into two orientations with respect to the adapter sequences. Comparing the sequences of two chain orientations makes it possible to reconstruct the original sequence before bisulfite treatment with the help of standard software and newly developed software written in C and described here. We provide a proof of concept via data obtained from almond (*Prunus dulcis*). Example data and a detailed description of the complete software pipeline starting from the raw reads up until the final differentially methylated cytosines are given in Supplementary Material making this technique accessible to non-expert computer users. The adapter design showed in this paper should allow the use of a two restriction enzyme approach with minor changes in software parameters.

## Introduction

Contemporary understanding of epigenetics encompasses “the study of changes in gene function that are heritable and that do not entail a change in DNA sequence” (Wu and Morris, 2001). These changes comprise histone variants, posttranslational modifications of amino acids on the amino-terminal tail of histones, and covalent modifications of DNA bases (Dupont et al., 2009). Most research on epigenetics focuses on DNA methylation, because the covalent changes in DNA bases are relatively easy to investigate with modern sequencing technologies. As a result, the term “epigenetics” is sometimes used to refer exclusively to DNA methylation (Seymour and Becker, 2017).

DNA methylation is under genetic control via a complex network of DNA methyltransferase and DNA glycosylase genes (reviewed in Pikaard and Scheid, 2014), although the extent to which epigenetic variation is under direct genetic control is not clear at this moment (Richards et al., 2017). Epigenetic variation can be the result of ordinary developmental processes that are triggered by internal signals (constitutive), such as those that occur during seed development or fruit ripening (reviewed in Li et al., 2018), or that are the result of external factors (facultative) like biotic or abiotic stress (reviewed in Bräutigam and Cronk, 2018). Additionally, spontaneous epimutations occur and change the DNA methylation pattern in unpredictable ways (reviewed by Richards et al., 2017; Johannes and Schmitz, 2019). Changes in the methylation pattern can be associated with gene expression levels. Generally, DNA methylation is linked to gene silencing, which is especially important in the control of the activity of transposable elements (reviewed by Hosaka and Kakutani, 2018). While the methylation of transposable elements, promotors and transcriptional start sites results in lower gene activity, gene body methylation is typical for housekeeping genes, which are expressed constitutively (Zemach et al., 2010; Bewick and Schmitz, 2017). The role of gene body methylation is not clear, although its conservation in plant evolution (at least 400 Myr) (Zilberman, 2017) and its apparently universal occurrence in the animal kingdom (Zemach and Zilberman, 2010) suggest its relevance (Bräutigam and Cronk, 2018).

In some cases, changes in methylation can be directly linked to distinct phenotypes. A naturally occurring form of *Linaria vulgaris* Mill. with radial flower symmetry instead of the bilateral symmetry of the wild type is characterized by an extensively methylated Lcyc gene, which is transcriptionally inactive; the demethylation of this gene activates the gene leading to the wild-type phenotype (Cubas et al., 1999). Other phenotypes that could be directly related to the methylation state of epigenetic alleles are the late flowering phenotype of fwa mutants in *A. thaliana* (L.) Heynh (Soppe et al., 2000); inhibited tomato fruit ripening (Manning et al., 2006); and sex determination in melon (Martin et al., 2009).

In natural plant populations DNA methylation is highly variable in different species (Richards et al., 2017). However, the rate and evolutionary significance of epimutations in these natural populations is at present largely unknown (Richards et al., 2017). On the other hand, there are several studies that document a correlation of epigenetic marks and environmental factors. For example, Herrera et al. (2016) concluded that in *Helleborus foetidus* L. the epigenetic spatial structure is driven by a moderate to high heritability and responsiveness to local environments. In addition, Alvarez et al. (2019) found differential methylation in oil-exposed and unexposed populations of *Spartina alterniflora* Loisel.

One elegant way to study differences in DNA methylation between samples is based on bisulfite sequencing. This technique takes advantage of the fact that sodium bisulfite causes the deamination of cytosines, unless they are protected by methylation (Frommer et al., 1992). This results in an uracil residue, which is later converted into thymine by a PCR reaction using a compatible polymerase. Sites where a thymine is identified after a bisulfite treatment, but a cytosine is found in the untreated reference indicate an unmethylated cytosine, while sites with a cytosine in the bisulfite-treated DNA indicate a methylated cytosine. This method was first applied to individual genes, but with the introduction of Next Generation Sequencing (NGS) platforms, scientists became aware of the possibility of obtaining the methylation pattern of all cytosines of a given genome (e.g., Cokus et al., 2008; Lister et al., 2008). While whole genome bisulfite sequencing (WGBS) has many advantages when studying model organisms with a known genome sequence, it cannot be applied to non-model organisms without considerable effort to create a *de novo* whole genome sequence. Even if a high-quality reference genome is available, in the case of experimental designs that require a large amount of samples like those encountered frequently in ecological research, the cost of WGBS can reach amounts that are prohibitive, especially in species with a medium to large genome size (Paun et al., 2019). When trying to obtain a genome scan in the search for differential methylation in natural populations of non-model organisms, researchers therefore used other techniques based on the fact that there are isoschizomer pairs of restriction enzymes, one methylation-sensitive and the other methylation-insensitive using a variant of AFLP (MS-AFLP; McClelland et al., 1994).

Several NGS-based protocols like RADseq (Baird et al., 2008), GBS (Elshire et al., 2011) and derived versions [e.g., double digest RADseq (ddRADseq), Peterson et al., 2012] are used to reduce the complexity of genomes by using restriction enzymes in order to obtain well-defined fragments. Barcoded adaptors make it possible to mix many specimens after the initial restriction-ligation steps into one sequencing lane, drastically reducing the cost per sample. The software pipelines developed to be used with this type of data like Stacks (Catchen et al., 2013) make it possible to work with species with known genome sequences but also with non-model taxa with no reference genome. Researchers interested in the bisulfite sequencing of non-model species became aware of the possibility of adapting the RADseq and GBS protocols in order to obtain the methylation data of reduced genome libraries in the absence of a reference genome. As a result of these efforts, three reduced representation bisulfite sequencing (RRBS) protocols were presented in 2016: epiRADseq (Schield et al., 2016), bsRADseq (Trucchi et al., 2016), and epiGBS (van Gurp et al., 2016). EpiRADseq uses a methylation-sensitive restriction enzyme (*Hpa*II, recognition site C↓CGG) together with an unsensitive restriction enzyme (*Pst*I, recognition site CTGCA↓G). Methylated *Hpa*II recognition sites are not cleaved and the corresponding fragments are absent from the resulting RRBS genomic library. The lab procedure follows essentially the standard ddRADseq protocol of Peterson et al. (2012) and only the computational analysis is adapted. While epiRADseq is not more expensive than ddRADseq, the disadvantage of this method is the fact that it only gives information about the methylation state of the *Hpa*II cut site, but not of the cytosines of the remainder sequenced fragments. The remaining two methods gain this information, but require the use of methylated adapters, which are much more expensive than unmethylated adapters. In the protocol of Trucchi et al. (2016), adapters are fully methylated. In the case of a project with 96 samples prepared in a library to be sequenced on one Illumina lane, 40 oligos with a total of approximately 436 methylated cytosines (depending on the barcode sequences) are needed. Additionally, in the absence of a reference genome, the protocol requires the sequencing of an aliquot of the library prior to the bisulfite treatment in order to build a “reference genome” with the aid of standard RADseq markers.

Although in their original epiGBS publication van Gurp et al. (2016) described the use of fully methylated adapters, hemimethylated adapters can be used (van Moorsel et al., 2019). In this case, the adapter strand whose 3’-end is ligated to the 5’-end of the genomic fragment is methylated while the protocol includes a nick translation step, which is used to repair the nicks between the 3’-end of the genomic fragments and the 5’-end of the unphosphorylated adapter sequences. The dNTP mix contains 5m-cytosine, which is used by the DNA polymerase I as an alternative substrate. As a result of the nick translation, the nick is repaired and the unmethylated cytosines in the adapter strands that are not ligated to the genomic DNA are replaced by 5 m-cytosine. But even so, 20 oligonucleotides with approximately 15 5-mC positions each (depending on the barcode sequence) are still needed, and the cost of the adapters can be higher than the Illumina sequencing of a paired-end library.

Protocols like GBS, RADseq, epiGBS, and bsRADseq use custom-made adapters instead of standard adapters supplied with kits. This is due to several restrictions given the specific conditions of these experiments. One major problem is that all these methods work with restriction enzymes and not randomly sheared DNA. As a result, all sequences start with identical base calls. But an equal per cycle composition of the first forward read bases is important in order to prevent phasing and pre-phasing detection errors (Kircher et al., 2011). In order to filter out PCR duplicates, adapters may be designed to integrate wobble positions. In the case of epiGBS it is convenient to introduce an unmethylated cytosine that can be used to calculate bisulfite conversion rates. But on the contrary, barcode indices must be methylated for epiGBS and similar protocols.

Here we present a variation of the original epiGBS protocol that uses unmethylated standard P1 GBS adapters presented by Poland et al. (2012) and requires only one hemimethylated P2 (common) adapter. This is a highly economical solution if standard GBS adapters are already available in a lab. If no standard GBS adapters are available, it is also possible to combine a high number of barcoded unmethylated P1 adapters with a low number of barcoded hemimethylated P2 adapters.

**FIGURE 1:**
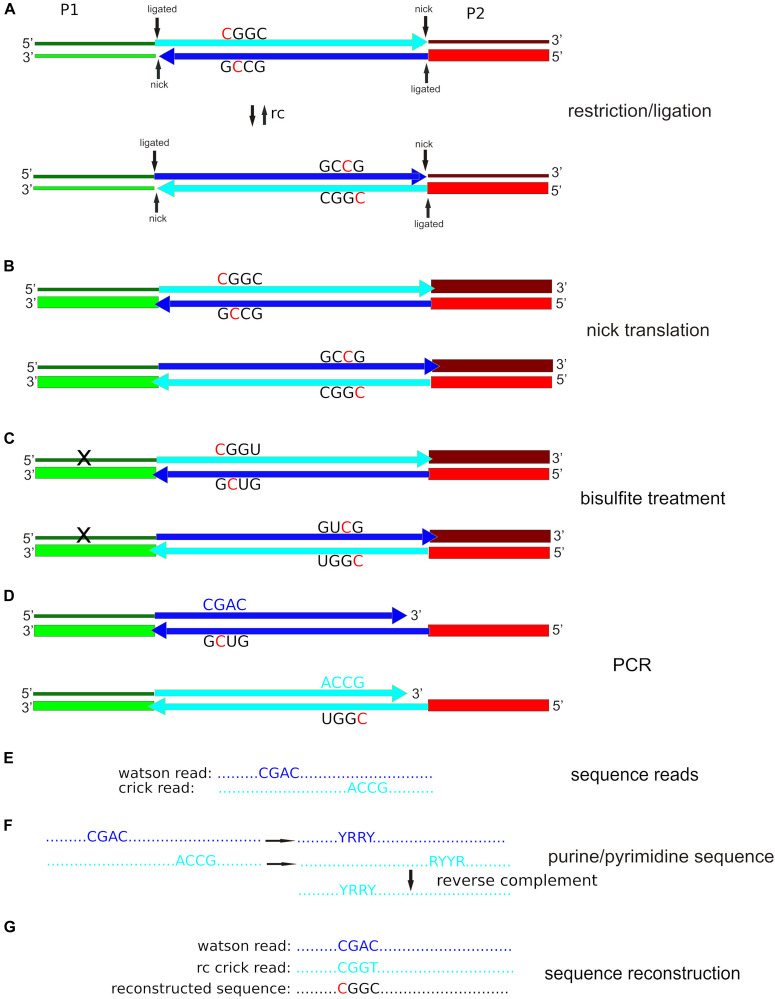
Simplified epiGBS scheme using our protocol with **Pst**I as an example. **(A)** Adapter P1 is a barcoded standard GBS adapter; adapter P2 is hemimethylated (only the lower strand has 5-methyl cytosine incorporated; indicated by a thick line). Both adapters are unphosphorylated at the 5′-termini. As a result, after the ligation reaction two nicks remain. The genomic DNA fragment is incorporated into two different orientations with respect to the adapter sequences, which are the reverse complement (rc) of each other. **(B)** After nick translation, the top chain of adapter P1 keeps unmethylated cytosines (thin line). The adapter sequences of the bottom chains are completely methylated (thick lines). **(C)** The bisulfite treatment converts cytosine to uracil unless the cytosines are protected by methylation. The top chain of adapter P1 contains a high number of converted unmethylated cytosines. **(D)** During the PCR step, uracil is read as thymine by a specially engineered polymerase. **(E)** Illumina sequence reads correspond to the complement of the bottom chain. **(F)** The software codifies DNA bases as either purines (R) or pyrimidines (Y). The program takes one arbitrarily defined Watson purine/pyrimidine sequence and tries to find the corresponding Crick sequence with an identical reverse complement purine/pyrimidine sequence. **(G)** If the software finds a Watson/Crick sequence pair, it compares the original Watson sequence with the reverse complement of the original Crick sequence. A cytosine in one sequence and a thymine in the other sequence indicate that there was an unmethylated cytosine in the original sequence. Two cytosines indicate a methylated cytosine in the original sequence, a guanine, and an adenine indicate a guanine with an unmethylated cytosine in the opposite strand in the original sequence and two guanines indicate a guanine with a methylated cytosine in the opposite strand of the original sequence.

We describe the necessary software tools – a combination of existing programs like Stacks (Catchen et al., 2013) or USEARCH (Edgar, 2010) and newly designed software for reconstructing the original sequence of the bisulfite-treated fragments. The reconstructed fragments are then joined into a mock genome. The mock genome can then be used with standard software tools like Bismark (Krueger and Andrews, 2011) and methylKit (Akalin et al., 2012) in order to extract methylation information and identify differentially methylated cytosines. Figure 1 explains the rationale behind our method. Detailed instructions on the use of the new programs together with preexisting software are given in the supplementary attached document. The instructions are presented in a way that is accessible for non-expert users with short shell-scripts that can easily be adapted to the specific conditions of different projects. Precompiled versions of the newly written programs (Linux operating system) and example data files are available for download. The instructions include detailed comments on the use of the different components of the software pipeline and how to change parameters if interested scientists want to use adapters different from those shown here or if other sequencing parameters (for example changed barcodes or enzymes) and/or read lengths are used.

## Materials and Methods

### Plant Material and DNA Extraction

We analyzed DNA from two almond [*Prunus dulcis* (Mill.) D.A. Webb] cultivars (cv. “Desmayo Largeta” and cv. “Penta”) at early and late stages during dormancy release (Prudencio et al., 2018). The DNA was extracted from a pool of 10 flower buds according to the protocol of Doyle and Doyle (1987). We performed the DNA extractions independently in two consecutive years. The DNA concentrations of the samples were measured in a Qubit 2 fluorometer and then adjusted to 20 ng/μl. The DNA extractions were stored at −80°C until use.

### Adapter Design

The design of the adapters is the essential difference of our protocol in comparison with other variants of epiGBS. The sequences of the barcoded P1 adapters correspond to standard GBS adapters and were taken from Poland et al. (2012). Their sequences are given in Table 1. The P1 adapters are completely unmethylated. The P2 adapter (see Table 2) was designed for this study. The upper strand is completely unmethylated. The P2 adapter carries five wobble positions, which can be used to eliminate PCR clones (Kebschull and Zador, 2015). If the raw data show an abnormally high number of duplicates, they can be filtered out with the help of the clone_filter module of Stacks, for example (Rochette and Catchen, 2017). Additionally, there is a 5 bp stretch that can be replaced by a barcode if necessary. All cytosines of the bottom strand of the P2 adapter are methylated with the exception of the cytosine in the *Pst*I overlap. As a result of the bisulfite treatment and the final PCR amplification, this position should be converted to thymine. The efficiency of the bisulfite treatment can be calculated as the number of converted cytosines/total number of cytosines at this position. If using other enzymes and/or adapters, an unmethylated cytosine should be integrated in the bottom P2 adapter strand outside the barcode region to guaranty the possibility to calculate the cytosine conversion rate. Both P1 and P2 adapters are designed to avoid the reconstitution of the restriction enzyme cut site after the ligation of the genomic DNA fragment to the adapters. The adapter sequences can be changed without any problems to adjust them to other enzymes or to implement specific desired characteristics like wobble bases in the P1 region or other barcodes. Supplementary Figure 1 shows a general scheme for this purpose. Special care should be taken when designing new barcodes. There are several points that require attention and the use of a GBS barcode generator like the GBSX barcode generator (Herten et al., 2015) is advisable. Recommendations on the design of new P1 and P2 adapters are given in Supplementary Figure 1.

**TABLE 1:** Sample identification, barcode, and adapter sequences for the top and bottom strand of the barcoded P1 adapters.

Sample	Barcode	Adapter sequence top 5′ ->3′	Adapter sequence bottom 5′ ->3′
AlDA1	CATCTGCCG	cacgacgctcttccgatctCATCTGCCGtgca	CGGCAGATGagatcggaagagcgtcgtg
AlDA2	GGACAG	cacgacgctcttccgatctGGACAGtgca	CTGTCCagatcggaagagcgtcgtg
AlDB1	ATCTGT	cacgacgctcttccgatctATCTGTtgca	ACAGATagatcggaagagcgtcgtg
AlDB2	AAGACGCT	cacgacgctcttccgatctAAGACGCTtgca	AGCGTCTTagatcggaagagcgtcgtg
AlPA1	GAATGCAATA	cacgacgctcttccgatctGAATGCAATAtgca	TATTGCATTCagatcggaagagcgtcgtg
AlPA2	TAGCAG	cacgacgctcttccgatctTAGCAGtgca	CTGCTAagatcggaagagcgtcgtg
AlPB1	ATCCG	cacgacgctcttccgatctATCCGtgca	CGGATagatcggaagagcgtcgtg
AlPB2	CTTAG	cacgacgctcttccgatctCTTAGtgca	CTAAGagatcggaagagcgtcgtg

*The sample code consists of the following elements: Al, Almond; *Prunus dulcis*; “D” or “P,” cultivar “Desmayo Largueta” or “Penta”; “A” or “B,” early or late in flower bud dormancy breaking, respectively; and “1” or “2,” year one or two of the experiment. The barcodes and adapter sequences are taken from Poland et al. (2012). The barcode part of the adapter sequences is given in upper case letters. Sample identification, barcode, and adapter sequences for the top and bottom strand of the barcoded P1 adapters.*

**TABLE 2:** Sequences of the P2 (common) adapter.

Adapter	Sequence 5′->3′
cre-epiGBS P2 top strand	*C*AGTTHHHHHagatcggaagagcggttcagcaggaatgccgag
cre-epiGBS P2 bottom strand	t5gg5att55tg5tgaa55g5t5tt55gat5tDDDDDAA5T*G*TG**C**A

*The top strand sequence does not contain methylated cytosines. In the bottom strand, all cytosines with the exception of the last one (corresponding to the *Pst*I overhang) are methylated (given as “5” instead of “C”). The unmethylated cytosine near the end of the bottom strand can be used to calculate the conversion rate achieved with the bisulfite treatment. Lower case letters indicate sequence parts corresponding to Illumina specifications. HHHHH and DDDDD are wobble positions that make it possible to filter out sequencing PCR replicates. The first five bases of the top strand and the AA5TG stretch in the lower strand can be replaced by a barcode sequence if combinatorial barcodes are used. Underlined parts belong to the enzyme-specific recognition site. In the case of *Pst*I, the first base (in italics, here C in the top strand and G in the bottom strand) should not be set to G in the top strand and C in the bottom strand in order to avoid the reconstitution of the *Pst*I cut site. The 3’ most C (in bold) of the bottom strand is unmethylated. This C should be converted into T after the bisulfite treatment and amplification with Kapa Uracil + polymerase. The bisulfite conversion rate can be calculated based on this position.*

### Restriction, Ligation, Bisulfite Treatment, and PCR Amplification

All these steps essentially followed the protocol previously described by van Gurp et al. (2016). Boquete et al. (2020) give a detailed description of the protocol with many useful hints for scientist aiming to implement these methods. The first step consists in the restriction of the genomic DNA and adapter ligation (Figure 1A). The important difference is that in our protocol the genomic fragments are integrated necessarily in two orientations with respect to the P1 and P2 adapters (Figure 1A) whereas in van Gurp et al. (2016) this is not the case. For this step 10 units of *Pst*I-HF (NEB, Ipswich, MA, United States) were added to cut 200 ng (20 μl) of genomic DNA in a 30 μl final volume in 1× CutSmart buffer. Reactions took place overnight at 37°C. The next morning, a mix of 1 μl barcoded P1 adapter (1 mM), 1 μl P2 adapter (1 mM), 1 μl T4 DNA Ligase (NEB, Ipswich, MA, United States; 400 units/μl), 0.4 μl ATP (Thermo Scientific, Alcobendas, Madrid, Spain; 100 mM), 1 μl of CutSmart buffer and 5.6 μl of water were added to each sample to reach a volume of 40 μl. The samples were then incubated for an additional 3 h at 22°C. After adapter ligation, the DNA samples were pooled, which was followed by a cleanup and concentrating step with the help of GeneJet Gel Extraction and DNA Cleanup columns (Thermo Fisher Scientific, Alcobendas, Madrid, Spain). The final elution volume was adjusted to 23 μl.

Because adapters are not phosphorylated, a nick remains between the 3’ terminus of the genomic fragment and the 5’ terminus of the adapters (Figure 1B). This nick is closed with the help of DNA polymerase I. Due to the 5’-3’ exonuclease activity of DNA polymerase I the nick repair not only closes the nick between the 3’ terminus of the genomic fragment and the unphosphorylated 5’ terminus of the adapter, but the complete adapter strand is replaced (van Gurp et al., 2016). This fact is used by the improved version of the epiGBS protocol (van Moorsel et al., 2019) to incorporate 5 methyl-cytosine into the adapter. To this aim, 19.25 μl of the cleaned digestion/ligation mix were incubated for one h at 15°C with 2.5 μl 5-mC-dNTP mix (10 mM, Zymo Research, Irvine, CA, United States), 2.5 μl NEB buffer 2 and 0.75 μl of DNA polymerase I (NEB, Ipswich, MA, United States; 10 units/μl). As a result of this step, three of the four adapter strands are methylated (Figure 1B).

The nick translation is followed by the bisulfite treatment (Figure 1C). We used the EZ DNA Methylation-Lightning Kit (Zymo Research, Irvine, CA, United States) following the protocol provided with the kit. At the end of the treatment, DNA was eluted in a volume of 10 μl and used directly for PCR amplification. At the end of this step, all unmethylated cytosines are converted to uracil. It is important to note that this is the case of the adapter sequence that was ordered unmethylated and not replaced in the course of the nick translation (upper left in Figure 1A).

The next step is the PCR amplification (Figure 1D). Four independent reactions of 25 μl each were set up. Each reaction included 2 μl of template DNA, 12.5 μl of Kapa HiFi HotStart Uracil + ReadyMix (Kapa Biosystems, Wilmington, MA, United States), 1 μl of Illumina PE-PCR primer 1 (5′-AATGATACGGCGACCACCGAGATCTACACTCTTTCCCTA CACGACGCTCTTCCGATCT-3′; 10 μM), 1 μl of Illumina PE-PCR primer 2 (5′-CAAGCAGAAGACGGCATACGAGATC GGTCTCGGCATTCCTGCTGAA-3′; 10 μM) and 8.5 μl H_2_O. Cycling conditions were set to an initial denaturation at 95°C for 3 min, followed by 20 cycles of 98°C for 10 s, 65°C for 10 s, 72°C for 30 s, and a final extension at 72°C for 5 min. Because the upper left adapter sequence (according to Figure 1) is changed by the bisulfite treatment, only the lower strands are amplified exponentially (Figure 1D). But because the genomic fragments are integrated in the two possible orientations with respect to the adapters (Figure 1A), it is possible to obtain the sequence information for both strands (Figures 1E–G; see section “Data Analysis”).

Before submitting the genomic libraries to the sequencing service, it is necessary to eliminate very small (primer dimers, if present, or very short genomic fragments) and too large DNA fragments. We used the MagJet NGS Cleanup and Size Selection Kit (Thermo Fisher Scientific, Alcobendas, Madrid, Spain) with an initial binding mix volume of 400 μl for an average desired DNA fragment length of 300 bp (=approx. 200 bp insert). Due to budget restrictions the almond library was mixed as 1/12 part with samples of another independent project. As a consequence, coverage of the almond sequencing is the same as would be expected in a 96-plex experimental design. The library was sequenced by Macrogen on an Illumina 2500 machine (2 × 100 PE option).

### Data Analysis

The data analysis is designed to build a catalog of the genomic fragments with the help of Stacks v2.4 (Catchen et al., 2013). In this catalog, the sequences that correspond to the same fragment but in the opposite orientation are separated in independent entries as Stacks is not designed to identify reverse complements. Furthermore, the sequences obtained from the both strands of the genomic DNA are not identical after reverse complementation because of the effect of the bisulfite treatment. Therefore, after catalog construction with the help of Stacks, custom designed software converts the original sequences to purine-pyrimidine sequences (Figure 1F). Because bisulfite treatment converts a pyrimidine (cytosine) to another pyrimidine (thymine), the reverse complements of reads with origin from opposite strands are identical when purines and pyrimidines are considered. Once identical reverse complements of the purine/pyrimidine sequences have been identified, we go back to the original sequences (Figure 1G). If one of the reads shows a thymine where the other shows a cytosine, the original state was an unmethylated cytosine, if both are cytosines, the original cytosine was protected from bisulfite action by methylation. In the supplement to this article we give detailed instructions on the use of the software pipeline. The provided material also contains shell scripts that can easily be adapted to user cases and then pasted into terminal windows for direct use.

In detail, the library was demultiplexed using the Stacks v2.4 component “process_radtags” (Catchen et al., 2013). It is important to use the “disable_rad_check” option, because the bisulfite treatment affects the *Pst*I recognition site. The sequences were then shortened by first eliminating the *Pst*I overhang of forward and reverse reads and truncating the sequences to 86 bases. As a consequence, all sequences across all samples are of the same length, independently of the length of the used barcode sequence, which simplifies the design of our own software. This was done using the “–fastqfilter” function of USEARCH v10 (Edgar, 2010) with the options “-fastq_trunclen 86” and “fastq_stripleft 4” for the forward reads and “-fastq_trunclen 86” and “fastq_stripleft 14” for the reverse reads. For other enzymes and/or read lengths, these parameters should be changed (see detailed information in the Supplementary Material). In both cases, it is mandatory to set the “–threads” option to one, because the default setting of “–threads” changes the order of reads in the output in an unpredictable manner on multicore systems, and as a result, forward and reverse reads no longer match if more than one thread is used. The resulting sequence pairs were then joined using “usearch –fastq_join –join_padgap ATATATAT – join_padgapq IIIIIIII” options. The resulting combined sequence consists of the two original reads separated by an artificial ATATATAT sequence, which is assigned a quality score of “IIIIIIII.” The joined sequences were then quality-filtered by “usearch –fastq_filter –fastq_maxee_rate 0.01.” This step eliminates sequences with a ≥ 0.01 probability of errors per base. The reads were aligned per sample into exactly matching stacks with “ustacks” with the default settings, and a catalog was built with “cstacks” (“-n 4” to allow 4 mismatches between sample loci). It is important to note that the output files of “cstacks” from Stacks v1 are organized in a different manner than those of Stacks v2. As a consequence, catalogs obtained with Stacks v1 are not compatible with our pipeline. The sequences of the catalog were read by the newly designed “creepi” program, which reconstructs the original sequences of each GBS locus before the bisulfite treatment and stiches them together to form a mock genome, which unifies the potential thousands of fragment sequences in one file for easier handling in the following steps. This rationale is similar to the one used in the GBS-SNP-CROP pipeline (Melo et al., 2016) and the bsRADseq software pipeline (Trucchi et al., 2016). Additionally, “creepi” eliminates the padgap part of the joined sequences, and if the forward and reverse reads overlap, the overlapping region is removed as well (merging).

“Creepi” also outputs a file with the individual sequences included in the mock genome together with the position of their boundaries in the mock genome and a plain fasta file with the individual sequences. The names given to the individual sequences are the line number corresponding to the first sequence that allowed the reconstruction of the original sequence. This feature allows tracing back the reconstructed sequence to the catalog.

The original sequence reads were mapped to the mock genome, and the cytosine methylation states were determined with the help of Bismark v0.19.0 (Krueger and Andrews, 2011) with the default settings, with the exception that the “–non-_directional” option was used. The correlation between samples and differentially methylated sites were identified by methylKit 1.4.1 (Akalin et al., 2012). The difference of the “getMethylDiff()” function was set to 25 and the qvalue to 0.01. The second column of the output contains the positions of the differentially methylated cytosines. This information can easily be extracted to a file with the help of an R script given in the Supplementary Material, which can be used together with the fragment file produced by cre-epiGBS to identify the original fragments where these positions are located. We present a program (seek_fragments) that is designed to extract this information.

**FIGURE 2:**
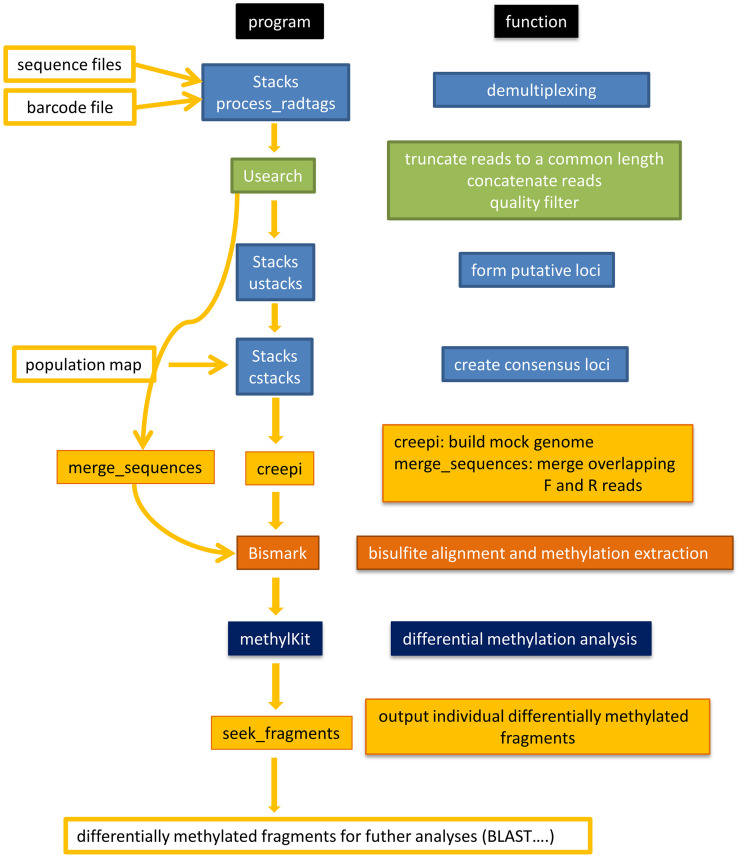
Flowchart to illustrate the software pipeline. A selection of freely available software (Stacks, USEARCH, Bismark, methylKit) and own software (creepi, merge_sequences, seek_fragments) was used. The freely available software was chosen for its ease of installation and good documentation. The function is specified for each program.

Figure 2 shows a flowchart of the software pipeline with the individual programs and their basic function.

We used SimRAD 0.96 (Lepais and Weir, 2014) to calculate the expected number of *Pst*I fragments of the *P. dulcis* Texas genome v2.0 (available at https://www.rosaceae.org/analysis/295) in given size ranges and to obtain the sequences of these fragments. We then searched a custom BLAST database constructed with the mock genomes obtained with our pipeline for homologous sequences.

## Results

With the modified adapters, we obtained 5,252,208–8,365,052 reads for the individual samples. After quality filtering, 2,055,858–3,494,318 joined sequences were retained, which means that 77.8–85.8% of the reads passed the quality filter. An initial test showed a low number of PCR clones (<1%), so filtering them out was therefore deemed unnecessary. The final mock genome consisted of 3,109 fragments. 2,467 of them showed homology with the *P. dulcis* reference genome. Of the homologous fragments, 1,813 produced one hit and the remaining fragments up to a maximum of five hits against the *P. dulcis* genome. Fragments that did not show homology with the *P. dulcis* genome were not filtered out, because 15% of them showed homology with other Rosaceae sequences in public databases, and no match was found for 45% of them (*E*-value cut-off: 0.001). The vast majority (36%) of the remaining non-*P. dulcis* fragments belonged to fungi, mainly the yeast-like *Pseudomicrostroma glucosiphilum* T. Kij. & Aime (Basidiomycota) and *Aureobasidium* ssp (Ascomycota). Under most scenarios fragments that do clearly not belong to the target organism can be filtered out easily at the end of the pipeline. The resulting mock genome had a length of 662,459 bp, which means 0.28% of the 246 Mbp *P. dulcis* genome (Sánchez-Pérez et al., 2019).

With SimRAD and the available *P. dulcis* reference genome, we calculated that 1,438 *Pst*I-*Pst*I fragments are expected to be in the range of 150–250 bp, 2,925 in the range of 100–300 bp and 4,179 in the range of 100–400 bp. We built a BLAST database of the mock genome with the makeblastdb order (Altschul et al., 1997) and searched the database against the expected sequences of the *in silico Pst*I digestion of the almond genome. Within the mock genomes, we found homologous sequences to these expected sequences in 1,193 fragments in the 150–250 bp range (83.0%), in 2,526 of the expected 2,925 fragments in the 100–300 bp range (86.4%) and in 3,266 of the expected 4,179 fragments in the 100–400 bp range (78.2%). The fragments created *in silico* produced always exactly one hit in the mock genome. The two BLAST searches together indicate that our pipeline merges on occasion different genetic loci with identical or nearly identical sequences as one locus of the mock genome. This fact also explains why the number of *in silico* fragments that hit the mock genome is higher than the number of fragments of which the mock genome consists. Increasing the parameter *-n* when building the catalog with “cstacks” might lead to more merged loci in the mock genome but lowering *-n* has the effect of considering fragments with different methylation states as distinct loci.

We then ran the Bismark alignment step with the obtained mock genome as genome file and extracted the methylation information. Of a total of 21,425,884 fragments 13,209,275 (61.65%) gave unique best hits and 115,465 did not map uniquely. We achieved a high coverage with mean values ranging from 211 to 342 reads per base with a minimum of 10 reads. The percentage of cytosines methylated in the CpG context was 31.23% (±0.53% SD) in the “Desmayo Largueta” cultivar and slightly lower at 30.00% (±0.77% SD) in the “Penta” cultivar. In the CHG context, 1.28% of the cytosines were methylated in both cultivars. The methylation state in the CpG context between biological replicates (same cultivar, same flowering stage, but different year) was highly correlated in all four cases (Pearson’s *r* = 0.99), estimated by methylKit (Akalin et al., 2012). At this stage, 98.3% of the fragments with differentially methylated cytosines could be mapped against the *P. dulcis* genome in a local BLAST 2.8.1 (Altschul et al., 1997) search, while the remaining 1.7% could be matched against other Rosaceae sequences deposited in the GenBank. No other fragments with differential methylation were detected. Details on the biological importance of our results are published elsewhere (Prudencio et al., 2018). In summary, most of the observed differential methylation corresponded to differences between cultivars, but in ten fragments it was correlated with flowering stage.

With a small dataset like the one presented here the whole pipeline can be run in one day. Our developed programs require very limited computer resources. The creepi program, which makes the most extensive calculations of our own software occupied 56.2 MB of computer memory and needed 20.3 ± 0.7 s (*n* = 5) execution time on an Intel Xeon E5-2630 v4 (2.2 GHz) machine with 125.8 GB RAM. However, it should be noted that the execution time of creepi grows with the square of entries in the catalog produced by Stacks.

## Discussion

The recent papers of van Gurp et al. (2016) and Trucchi et al. (2016) make it possible to use NGS in studies of DNA methylation in non-model organisms. Based on these protocols, studies that involve a high number of individuals, like population genetics studies, are feasible at a reduced cost in the absence of available genome sequences in public databases. Nevertheless, the costs for hemimethylated adapters remain high and can be greater than the costs for Illumina sequencing in small-scale projects that use only a few sequencing lanes. In the absence of a reference genome, the protocol of Trucchi et al. (2016) also requires the parallel sequencing of non-bisulfite treated samples in order to reconstruct a reference for methylation calls. This is due to the fact that the software pipeline compares the bisulfite treated fragments to an untreated reference and when it encounters a thymine in the treated fragment where there is cytosine in the reference, it concludes that there was an unmethylated cytosine in the genome. Cytosines in the sequence of treated fragments correspond to methylated cytosines in the genomic DNA. The need of a reference was eliminated by van Gurp et al. (2016) using the information available in the complementary strands of the genomic DNA, looking for G-T and G-C base pairs.

Our protocol requires only one hemimethylated P2 adapter, while the barcoded P1 adapters are unmethylated. The unmethylated P1 adapters are hemimethylated by a nick translation reaction. As shown in Figure 1, it is possible to reconstruct the original sequence of a bisulfite-treated GBS fragment in the absence of a reference genome if the forward sequence and the reverse complement of the bisulfite treated DNA are available in a way similar to the original epiGBS protocol. The necessity of additional sequencing of untreated DNA required in other protocols like bsRADseq is therefore eliminated. If standard GBS adapters are available, the two DNA oligonucleotides of the P2 adapter (one methylated) are the only ones that need to be newly synthesized. If there are no GBS adapters already available in the lab, the least expensive solution is to combine a high number of barcoded unmethylated P1 adapters and a low number of barcoded hemimethylated P2 adapters and calculate the cost for the adapter combination that reaches the minimum price for the desired number of samples. In the Supplementary Table 1, we show the calculation of the total cost of our method in comparison with published protocols for 96 samples. The savings are in the range of $2,000–$4,500 compared to the epiGBS protocol and $5,900–$8,300 compared to the bsRADseq protocol. The highest savings can be achieved when the lab already uses standard GBS barcoded P1 adapters. Another important factor is the price the manufacturers charge for each methylated cytosine, because there are huge differences between the different companies. The quantity of the oligos delivered by the manufacturers is sufficient for a high number of assays, so the cost advantage of our method per sample will be diluted in high throughput labs. As a result, our protocol is especially interesting for small labs or pilot studies with a low number of libraries to be sequenced.

We could reconstruct over 80% of the fragments in the 100–300 bp range. The percentage of fragments that can be reconstructed in other settings depends on several factors. The most important are genome size, the restriction enzyme used and the number of samples per sequencing lane. The *P. dulcis* genome is relatively small (aprox. 246 Mb, roughly double the size of the *A. thaliana* genome), and around 2,925 *Pst*I fragments are expected in the 100–300 bp size range. *Pst*I is a six-cutter restriction enzyme, and as a result, using for example a 4.5-cutter restriction enzyme like *Ape*KI frequently used in GBS would probably drastically reduce the mean coverage per base if the number of samples was not adjusted accordingly. If whole genome data of organisms close to the species in question are available, the use of bioinformatic instruments like SimRAD (Lepais and Weir, 2014) can help to find good starting points for the design of a project and fix an appropriate number of samples per sequencing lane. In the absence of genome data, SimRAD can also be used to generate a random genome of a given length and a fixed GC content. *C*-values for many plant species, which can easily be converted into genome length in bp, are available, for example, at the Plant DNA *C*-values Database of the Royal Botanical Gardens at Kew (data.kew.org/cvalues/).

The choice of the restriction enzyme also has consequences with respect to the genomic regions that are of special interest. For example, *Pst*I has the recognition site 5′-CTGCAG-3′. This site is not affected by CpG methylation but by CHG (CTG; CAG) methylation. Nevertheless, CHG or CHH methylation can be detected in the fragments obtained with *Pst*I. But in *A. thaliana* at least, CHG methylation is spatially autocorrelated (Cokus et al., 2008; Becker et al., 2011), and methylation rates in these fragments could be underestimated if partially methylated *Pst*I recognition sites are present (van Gurp et al., 2016). Available data (Becker et al., 2011; van Gurp et al., 2016) suggest that this behavior might steer the obtained fragments away from repetitive regions like transposable elements, which show a higher incidence of CHG methylation and favor the targeting of coding regions and their vicinity, which are less prone to CHG methylation. This might explain in part the considerable differences found between CpG methylation and CHG methylation in our results, which are similar to those found by van Gurp et al. (2016) for several plant species using *Pst*I. Nevertheless, Alvarez et al. (2019), also using *Pst*I as restriction enzyme, found an only slightly lower CHG than CG methylation in *Spartina alternifolia*. The data of van Gurp et al. (2016) also show that the coverage of chromosomal regions (1 MB window) with a high methylation rate is low in *A. thaliana* with *Pst*I epiGBS data. Depending of the aim of a project, this might be an advantage (if coding regions are of major interest) or a disadvantage (if an equal representation of the whole genome is the aim of the project). Restriction enzymes combinations like *Csp*6I-*Nsi*I (recognition sites G↓TAC and ATGCA↓T) represent all three methylation contexts (CG, CHG, and CHH) equally well and might therefore be better suited for certain experimental setups (van Moorsel et al., 2019).

Another important factor to be considered with respect to the choice of the restriction enzyme(s) to use is the number of expected fragments in the targeted size range. For example, Sonah et al. (2013) calculated that in soybean, *Mse*I produces 9.5 million fragments, *Ape*KI 800,000 fragments and *Pst*I 100,000 fragments, but in the case of *Mse*I, many fragments are below 100 bp, and in the case of *Pst*I, a high percentage of fragments has a length of over 500 bp. As a result, the number of usable fragments varies largely as a function of the restriction enzyme used. Enzymes that produce a low number of fragments in the size range used by NGS sequencing (like *Pst*I) are appropriate for genotyping a moderate number of markers with a high multiplexing level and large genomes (Hamblin and Rabbi, 2014), while frequent cutters can be used if the study aims to produce a high number of markers with a low multiplexing level and in organisms with small genomes. Schmidt et al. (2017), for example, showed that combinations of *Msp*I with *Dpn*II or *Ape*KI resulted in a high genome coverage and high cytosine coverage. Although these authors do not specify the number of sequencing lanes they used on an Illumina HiSeq 2500 machine, from the available data it is evident that the multiplexing level in their study was low. It should also be taken into account that high coverage is desirable for the calculations necessary to identify differentially methylated cytosines. Software like SimRAD (Lepais and Weir, 2014) may help to make the best possible decision regarding the choice of restriction enzyme, coverage, and multiplexing level. Most real-world scenarios where epiGBS is applied will depend on a high number of samples in order to find significant signals, especially in the field of molecular ecology and evolutionary biology. In our case, although we used only eight samples, they occupied 1/12 of the entire library sent for sequencing. Therefore, the coverage we found is expected to correspond to a 96-plex experiment. The high coverage in our experiment with mean values clearly above 200 reads indicates that using *Pst*I in an organism with a relatively small genome like *P. dulcis* allows for high multiplexing. Alvarez et al. (2019), who used *Pst*I in *Spartina alterniflora* with a genome roughly seven times the size of the *P. dulcis* genome (Baisakh et al., 2009), processed 48 samples together.

If a reference genome is available, the fragments obtained by our lab protocol can be directly used as input for methylation extraction after the trimming of technical sequences (barcodes, wobble, etc.) and quality filtering. Therefore, epiGBS in its different variants might be an interesting option in organisms with known genome if a high number of samples is used and whole genome bisulfite sequencing is not cost-effective.

In comparison standard epiGBS protocol (van Gurp et al., 2016) there is no theoretical reason why our method should produce significantly different results when the same restriction enzymes are used. Our mock genome covers 0.28% of the 246 Mb almond genome, while van Gurp et al. (2016) covered 0.37% of the 135 Mb genome of *A. thaliana*. We recovered 86.6% of the theoretically expected *Pst*I fragments in the range of 100 – 300 bp, while in *Arabidopsis* 89% of the fragments in the range of 11 – 300 bp were found. We calculated a Pearson’s *R*^2^ of 0.98 between replicates while in *A. thaliana* this value was 0.95. Although other quality related indicators like less than 1% PCR clones, a high coverage of reads (mean value 211 – 342) similar read number for different samples (5,252,208–8,365,052 reads) and 80% of sequences passing the quality filter are adequate.

The method as presented here is limited to the use of only one restriction enzyme, but combinations of enzymes like *Pst*I-*Msp*I can be used if two sets of adapters are used. In the case of *Pst*I-*Msp*I, for example, this means that a set of unmethylated P1 adapters compatible with *Pst*I and another set compatible with *Msp*I are needed. The unmethylated P1 *Pst*I adapters are then combined in the restriction-ligation steps with hemimethylated P2 adapters with *Msp*I ends and the unmethylated P1 adapters with *Msp*I ends with hemimethylated P2 adapters with *Pst*I ends. In this case, the number of necessary adapters is double that of the one enzyme only case, but the cost should still be lower than that of the original epiGBS protocol.

The software is expected to work in the two-enzyme case as well, if adapters are adjusted and the software parameters are set accordingly (explained in the Supplementary Material). Nevertheless, the runtime is higher than in the case of a specially developed software, because in its present state the software does not take into account the strand information available under the two-enzyme scenario.

At the end of the pipeline presented here, information on differentially methylated cytosines is obtained. The fragments are exported in a fasta formatted file that can be used as input for other software packages like Blast2Go^1^ for the functional analysis of the datasets.

Computer programs that are complicated to use may have a deterrent effect on scientists who are interested in a biological problem but are no computer experts. We have made a considerable effort in trying to make the bioinformatic pipeline as straightforward to use as possible. We found that the third-party computer programs we use in our pipeline are very well documented with detailed manuals and easy to install. The newly designed software is explained in the supplement and some common pitfalls for less experienced computer users are mentioned. The supplement includes all the orders (shell scripts) that are needed to get the programs to work (third-party and new). We gave the data and scripts mentioned in the supplement to Ph.D. students without prior experience in epigenetics and average knowledge in other fields of bioinformatics and they were able to follow the steps with only the help of the instructions given in the supplement.

Summarizing, the most important differences of our method in comparison with other RRBS protocols are that epiRADseq uses only the information of one restriction enzyme cut site, while we use the sequence of the whole fragment. On the other hand, BsRADseq requires the parallel sequencing of an untreated and a bisulfite treated library and epiGBS needs hemimethylated adapters on both sites of the genomic fragments created by the restriction enzymes while with our method a hemimethylated common adapter is sufficient. As a consequence, our variation of the epiGBS protocol of van Gurp et al. (2016) has the advantage of a much lower cost associated with the purchase of methylated adapter oligos. Existing GBS barcoded adapters can be used in combination with a hemimethylated P2 adapter. These advantages are especially important for smaller laboratories with limited financial resources. The high correlation of the methylation data of the biological replicates (*r* = 0.99) shows the reliability of the data sets created by our method.

## Data Availability Statement

DNA sequence reads generated for this study can be found in the NCBI SRA (accession numbers SRX7526585–SRX7526592). All newly designed software and shell scripts available at https://github.com/olafumes/creepiGBS.

## Author Contributions

OW conceived the method, performed the lab work, developed the software, analyzed the data, and wrote the manuscript. ÁP supplied the samples, performed the lab work, analyzed the data, and contributed to writing the manuscript. EC-M and MN-L tested the software and contributed to writing the manuscript. PM-G and RR analyzed the data and contributed to writing the manuscript.

## Conflict of Interest

The authors declare that the research was conducted in the absence of any commercial or financial relationships that could be construed as a potential conflict of interest.

## Footnotes

1 https://www.blast2go.com
