# Supplementary material for: A Cost Reduced Variant of Epi-Genotyping by Sequencing for Studying DNA Methylation in Non-model Organisms
Source: Front Plant Sci. 2020 May 28;11:694. doi: 10.3389/fpls.2020.00694 (PMC7270828; doi:10.3389/fpls.2020.00694)
Supplement: Supplementary file 1 [file Data_Sheet_1.docx]

Supplementary Material

# Bioinformatic pipeline

In this annex we present a bioinformatic pipeline for non-expert computer users. In many cases there exists alternative ways to proceed. The only important points are that our creepi program expects a Stacks v2 catalog with equal length sequences that start with the sequences of the restriction cut sites as indicated in Supplementary Figures 2 and 3.

**Pipeline programs**

1. **Stacks** v2 can be downloaded from <http://catchenlab.life.illinois.edu/stacks/>. This suite of programs can be built on UNIX-like environments (Linux, Apple OS) and comes with detailed installation instructions. Note: it is important to work with Stacks v2 and not Stacks v1 for generating the catalog because the format of the output file changed and only the output of v2 is compatible with our creepi programm.
2. **USEARCH** v10 can be downloaded from <https://www.drive5.com/usearch/>. The program comes in a precompiled version. The 32-bit version is free, and the 64-bit version must be bought. There are versions for Linux, Apple OSX and Windows. If you want to use USEARCH without specifying the location of USEARCH every time or move USEARCH to the folder you are using at a given moment, you have to change the PATH variable of the shell. On many Linux systems, the bash shell is run. You can confirm whether your system uses the bash shell with the command ($ indicates the command prompt):

- $ echo $SHELL
- You should get something like
- /bin/bash
- You can then open a file called *~/.bashrc* with a text editor, for example with the command:
- $ nano ~/.bashrc
- and add the line
- export PATH=$PATH:/place/with/the/file
- at the end of the file. Save the file and the system should find your program.

1. **Bismark** v0.19.0 is basically a series of Perl scripts that can be downloaded from <https://www.bioinformatics.babraham.ac.uk/projects/bismark/>. Bismark itself depends on Bowtie or Bowtie2, and for BAM output, SAMtools is required. Bismark comes with an extensive user guide. Installation is straightforward. Essentially just extract the files. Bowtie can be downloaded from <http://bowtie-bio.sourceforge.net/bowtie2/index.shtml>. Indications for the installation of SAMtools can be found at <http://samtools.sourceforge.net/>.
2. **methylKit** is an R package and is part of the Bioconductor software packages. Instructions on how to install it can be found at <http://bioconductor.org/packages/release/bioc/html/methylKit.html>.
3. For this paper, we developed three C programs. The program **creepi** takes a stacks v2 catalog as input and outputs a mock genome, a fasta file of individual fragments reconstructed from the bisulfite-treated input and the same fragments with position numbers delimiting the beginning and end of each fragment in order to identify, at the end of the process, the fragments that individual differentially methylated cytosines belong to. The program **merge_sequences** looks for overlaps of forward and reverse reads the same way creepi does. This facilitates the alignment step of Bismark. Finally, **seek_fragments** takes a vector of positions of differentially methylated cytosines and the file of individual fragments with their beginning and end position numbers as input and outputs the fragments that contain differentially methylated cytosines in a fasta file. The fasta file can then be used to search databases for information on these fragments (e.g. BLAST search). Make sure that the scripts and programs are made executable with the command:

- $ chmod u+x *scriptname*

**Step-by-step example with shell scripts that can be used**

Below we provide a step-by-step example to show you how the pipeline works once all programs are installed. You can modify the scripts for your own data. We explain the rationale behind the parameter settings that depend on the type of data (e.g. read length, barcode length, adapter design) so that you should be able to make the necessary adjustments if you use for example 125 bp PE reads instead of the 100 bp PE reads we used.

For the scripts, we assume that USEARCH and Bowtie2 are in the PATH. If you have problems executing the scripts and programs, check that *y*ou are using the program names exactly as they are in the scripts. If this is not the case, change the script or the program names accordingly. A common error for people not familiar with LINUX terminals is to forget the ./ you have to place first to indicate that you are referring to a file placed in your present working directory.

The program **process_radtags** is used to separate the sequences according to their barcodes. It needs the directions where the two files with forward and the reverse reads are stored, the direction of the file with the **barcodes** and information regarding the name of the restriction enzyme used. Because the sequence of the PstI recognition site is changed by the bisulfite treatment, it is mandatory to deactivate the check of the restriction site with the --disable_rad_check flag. There are several options that in certain cases must be changed or that an advanced user might want to modify. For example, if you use combinatorial barcodes, indicate the --inline_inline flag.For more information, please refer to the stacks documentation. Let´s assume that the barcode file PstI_Barcodes_Almond.txt and the two read files Almond_1.fastq.gz and Almond_2.fastq.gz

are in the same working directory as the process_radtags_script.sh. Additionally, let´s assume that you have created a subdirectory with the name almond in your working directory to store the output of the process_radtags program. Open a terminal and move with the

$ cd command to the working directory and type in:

$ ./process_radtags_script.sh

and soon the output will be located in the almond subdirectory.

If clone_filter is used, it should be done after demultiplexing the data, but before truncating the sequences during the next step. Please note that clone_filter does not offer an –null_inline option for the position of the wobble sequence. Therefore, it is necessary to invoke the –inline_inline oligo sequence option. –oligo_len_1 can be set to 1and –oligo_len_2 should be set to 5. If you use our scripts, please take care that the output of clone_filter is named and placed in the folder expected by the next step.

The next step is to truncate all the forward and reverse reads to the same final length. It is also of **central importance** that the same number of bases is removed from the beginning of the forward reads and the end of the reverse reads with reference to the recognition site of the restriction enzyme. In our example, the barcodes used are of variable length (5bp – 10bp) and are already removed by process_radtags. The PstI recognition site has the sequence 5’-C|TGCAG-3’. The first cytosine has already been replaced by another base while adding the adapter to avoid reconstituting the recognition site after adapter ligation. This position is included in the barcode sequence. We want to remove the the PstI overhang of 4bp length, because at the site of the P2 adapter it is given by the adapter sequence and not by the genomic sequence. As a result, we first have to remove the four bases of the overhang from all forward reads and then truncate the sequences to a uniform length of 86 bp (100 read length – 10 max. barcode length – 4 PstI overhang). The adapters used here at the P2 terminal all start with 5bp wobble sequences followed by 5 bp that can be replaced by a second barcode if necessary. The fifth base is also used to replace the last guanine of the recognition site to avoid its reconstitution. This has to be taken into account when designing barcodes at this P2 site on your own. The PstI overhang adds 4 more bases that should be removed. As a result, by removing 14 bases from the left side of the reverse reads, all reverse reads will start with the last base of the PstI recognition site -the same as the forward reads- and have the same final length of 86. Just what we need!

In the case of a **two-enzyme approach**, remove the recognition site of both restriction enzymes completely and start the sequences with the first base after the recognition site. In the present form of the software, it is also necessary to truncate the sequences of all reads to the same final length, although a specifically designed software could work with different read lengths for enzyme one on the one hand and enzyme two on the other hand.

All this can be done in one step with USEARCH –fastq_filter. This program can first strip a number of bases from the left or right ends of DNA sequences and truncate the result to a given final length. The default behavior of USEARCH is to use more than one thread if the computer has sufficient cores, but this causes problems in our pipeline as we want to join forward and reverse reads at a later stage. If more than one thread is in action, the order of the forward and reverse reads of the output are no longer the same. It is therefore **important** to use the option -threads 1. If you want to use our scripts, move to the almond subdirectory and place a copy of script1.sh here. This shell script has all the instructions for USEARCH for the files. Make the script executable and create a subdirectory called short. Execute the script and the truncated sequences should appear in the short subdirectory.

We now want to stitch together the forward and reverse reads. This can be done with USEARCH -fastq_join. The two reads are separated by an arbitrary ATATATAT sequence (-join_padgap) and the quality score for these bases is set to I (-join_padgapq) If you want to use script2.sh,place a copy of this script in the short subdirectory and make it executable. Create a subdirectory called joined

and execute the script.

The next step is to check the quality scores of the resulting sequences. USEARCH offers a wide range of quality-filtering criteria that can be applied. Here we opt for the criterion to discard sequences if the number of expected errors per 100 bases is greater than 1. For more information, please consult the USEARCH manual. If you want to follow our example, place script3.sh in the joined directory and make it executable. Create a subdirectory called quality and execute script3.sh. The output is placed in the new subdirectory.

At this point, **ustacks** is used to build stacks of sequences that correspond to putative alleles and loci. The default value of -M, which indicates the maximum distance allowed between stacks, is 2 and low if we want to search for differentially methylated cytosines in each of the samples. We use –M 4 in our example script. Each sample has to be given an individual number as an identifier. To accelerate the action of ustacks, this program can be activated with the -p n flag were n Is the number of parallel threads that will be executed. Make sure that this number is not higher than the number of cores of your machine. For this example, we set –p to 2, which should not be a problem on any modern computer, and most users can probably use higher numbers. To continue with our example, place script4.sh in the quality directory and make it executable. Create a new subdirectory called stacks and execute script4.sh.

During the following step, a catalog of all loci of all samples is built with **cstacks**. In addition to the files created by ustacks, a **population map** is needed. For our purposes the population map is only important because it indicates the names of the input files to cstacks. -n indicates the number of mismatches between sample loci when the catalog is built. The default value is 1.The results are automatically stored in several files that start with batch_1. The file we are interested in is called catalog.tags.tsv and includes the consensus sequences of all putative loci. To get cstacks working, we place the file Al_Map in the stacks directory. Then we move one level up (to the quality directory), where we place script5.sh and make it executable. After the execution of script5.sh, catalog.tags.tsv should appear in the stacks subdirectory.

The program **creepi** takes a file with the format of the catalog built by cstacks. The consensus sequences are reverse complemented, and original sequences and reverse complements are converted into purine/pyrimidine sequences. The program then searches for pairs of purine/pyrimidine sequences where the original sequence of the first is identical to the reverse complement of the second. If the program finds a pair fulfilling this condition, the original sequence of the genomic fragment before the bisulfite treatment is reconstructed. The options are as follows:

-f: input file. The input file is the catalog built by cstacks. Please omit the .tsv extension. Mandatory.

-1: The sequence that remains of a restriction recognition site in cases in which the genomic fragment is shorter than the read length at the 3’-end of the forward read. If the enzyme has a degenerate recognition site, use one of the possibilities. Mandatory.

-2; -3; -4: If the enzyme has a degenerate recognition site, use this options to indicate additional recognition sequences at the 3’-end of the forward read. Optional.

-5: The sequence that remains of the restriction recognition site in cases in which the genomic fragment is shorter than the read length at the 5’-end of the reverse read (in the direction the sequence is shown in the Illumina files). If the enzyme has a degenerate recognition site, use one of the posibilities. Mandatory.

-6; -7; -8: If the enzyme has a degenerate recognition site, use this options to indicate additional recognition sequences at the 5’-end. Optional.

How to obtain the sequences for options -1 to -4 is explained in detail below.

-O: creepi looks for **o**verlapping forward and reverse reads. The default behavior is to first compare 15 bases at the 3’ terminal of the forward read with 15 bases at the 5’ terminal of the reverse read. If there is a perfect match, the 15 bases of the reverse read are cut out and the two reads are stitched together. If there is no match, in a sliding window approach the number of compared bases is increased until the end of the read is reached. If you want to use less stringent criteria for a match, a positive integer (<15) can be used with this option to indicate the number of mismatches allowed in the overlapping region. Optional, default = 0.

-M: The default behavior of the program when searching for identical purine/pyrimidine original sequences and reverse complements is to accept only perfect matches. The -M option indicates the maximum number of mismatches allowed.

**How to find the right values for options -1, -2, -3, -4, -5, -6, -7, -8**

Using the example PstI, the sequence of the recognition site is 5’-CTGCA|G-3’. But at the P1 site the first C is replaced by another base of the adapter and only the 5’-TGCA|G-3’ part remains of the original recognition site. At the P2 end, the guanine of the 3’ end is replaced by another base (we use a cytosine in our protocol). As a consequence, only the 5’-CTGCA|-3’ remains. But due to the specifics of our protocol, only the complements of these sequences survive (see Figure 1 of the main text and Supplemental Figures 2 and 3). These would be 3’-ACGTC-5’ at the P1 end and 3’-GACGT-5’ at the P2 end of the fragments. After bisulfite treatment and PCR amplification, the complements are converted to 3’-ATGTT-5’ and 3’-GATGT-5’. The sequence reads, which are in the direction given for the recognition site, should therefore be 5’-TACAA-3’ and 5’-CTACA-3’. As we have seen above, the sequence reads are already trimmed in a manner that the cut site at the beginning and end of the fragments is removed with the exception of the last base (from the nearest adapter sequence). But if the genomic fragment integrated between the adapters is shorter than 86 bp, we would expect to find the “CTACA”- sequence somewhere before the end of the sequence read and the “TACAA” sequence at exactly the same distance from the P2 extreme of the merged sequence. This information is used to cut “dovetailed” reads (= completely overlapping reads in which the read sequence reaches the opposite adapter) and eliminate all parts that do not belong to the genomic fragment. To make this possible, the options -1 and -3 are needed. -1 is used to pass the sequence we would expect somewhere before the end of read one after trimming in the case of a genomic fragment shorter than 82 bases in our example. In the case of PstI, this would be “CTACA”, and -3 would be “TACAA”. Options -2 and -4 are not mandatory. They are used in the case of enzymes like ApeKI, which have ambiguous recognition sites (in the case of ApeKI 5’-G|CWGC-3’, W = A or T ). The resulting options are -1 ACAA -2 ACTA -3 CAAC -4 CTAC (the order within each extreme does not matter). To illustrate this matter, see the Supplemental Figure 2.

To get creepi working, place a copy in the quality directory. You also need a copy of “creepi_script.sh” in this directory and the copy should be made executable. The script only uses the options -1, -5 and –f, which are mandatory. Execute the script. The output will be placed in the stacks directory.

**Output of creepi**

creepi produces several outputs. The base name is recycled from the input name. The first output is a “mock genome” in fasta format (input_file_name_mock_sequence.fa). All reconstructed sequences are stitched together to form the mock genome. The second file is a fasta file with the individual fragments (input_file_name_fragments.fa). The names of the sequences are the numbers in the order they are stitched together in the mock genome. The third file is composed of the fragment with the beginning and end positions of each fragment (input_file_name_fragment_numbers.txt). This file is used later to identify fragments with indications of differentially methylated cytosines. Finally, the forth output file is similar to the input file, but with the addition of information like reverse complements of the original sequences, purine/pyrimidine sequences of the original sequences and the reverse complements and the reconstructed sequence of the fragments (input_file_name_out.tsv).

**creepi in a two-enzyme approach**

In the case of a two-enzyme approach, you need to figure out the sequences of the recognition sites of the both restriction enzymes as described above. Then take the lowest number of remaining bases of the two cutsites and truncate the sequence for the second, longer side accordingly. In the case of PstI and ApeKI, five bases remain at the PstI recognition site and four at the ApeKI recognition site. Therefore, use the innermost (close to the genomic fragment and away from the nearest adapter sequence) four bases of the PstI site and all four bases of the ApeKI site. Parameters -1 to -4 are used for the sequence, which is expected to be close to the 3’ end of the forward reads, and parameters -5 to -8 for the sequence which is expected to be close to the 5’ end of the reverse reads (already reverse complemented, to be in the orientation of the forward reads). To illustrate this matter, see the Supplemental Figure 3.

**merge_sequences**

This program merges sequences the same way creepi does, but it uses the the fastq datafiles that were used as input for ustacks. The options are as explained for creepi. The .fq extension of the input file must be omitted. The output are two files of the merged sequences, one in the fq format (with quality scores) and the other in plain fasta format (input_file_name_merged.fa and input_file_name_merged.fq). Quality scores are not adjusted for the overlapping regions. We take only the value of the forward read. This step is done with the aim to have no discrepancies between the mock genome and the individual fragments in the Bismark alignment step. To use this program, put a copy in the quality directory along with the script_merge_sequences.sh file. The quality directory should still contain the read files that you obtained after the quality filtering. If you followed all our prior scripts, these should all terminate with _joinedq.fq. Please use the filenames without the .fq extension as input, because merge_sequences will append this extension on its own. When you execute the script, the output files will have the same base name as the input files with _merged.fq and _merged.fa appended. The output will be placed in the folder where the infile is located.

**Bismark**

With the output of creepi and merge_sequences, we can start **Bismark**. Here we give a very basic outline that should work, but Bismark offers a lot of options that can be consulted in the manual.

First we need to run the Bismark genome preparation. To do this, copy the output of merge_sequences in the directory where the Bismark programs are placed (or place the Bismark programs in the directory with the merge_sequences output. Then create a new subdirectory named genome and move the mock genome to this subdirectory. Then just execute

$ ./bismark_genome_preparation ./genome.

This order will prepare the genome sequence in the genome directory for the next step.

Regarding the Bismark alignment step, it is important to use the **--non_directional** flag. An example would be

$ ./bismark --non_directional --genome ./genome ./your_file_name

If Bowtie is not in the PATH, you have to indicate to Bismark where to find it.

Finally, we need to invoke the methylation extrator of Bismark with ./bismark_methylation_extractor --bedGraph --cytosine_report --genome_folder ./genome bismark_output_bt2.bam.

If you use this line, make sure that your genome data is in a subdirectory named genome.

All of these steps are prepared for our example in the Bismark_script_complete.sh file. Move this script to the directory where the Bismark programs and the output files of merge_sequences (with terminations _joinedq_merged.fq) are located. Within this directory, create a file named genome and place a copy of the mock genome there. The pipeline will produce several outputs, which can be found in the present working directory.

Finally, the R package **methylKit** can be used to identify differentially methylated cytosines. methylKit can use Bismark coverage files as input in different methylation contexts and calculate different statistics referring to individual input files like methylation statistics and coverage statistics. A very important point is that it is possible to calculate the correlation between different samples and to finally identify differentially methylated cytosines. From the results table, it is possible to extract the position of the cytosines with reference to the mock genome.

With our example, we will use an R script (methylKit_R_script.R), which shows some functions of methylKit like getCoverageStats() and makes it possible to extract hyper-methylated and hypo-methylated cytosine positions when almond samples of the two varieties ‘Desmayo Largueta’ (with D in their file name) and ‘Penta’ (with P in their file name) are compared. To define this grouping, the “treatment” parameter of the methRead() function is used. In our example, the CpG reports are used, but it is possible to use other methylation contexts if you like. When you want to try our script, make sure to **load the methylKit package** and to **set the working directory** to the place where the CpG reports are situated. Also make sure that the file names of our script are exactly the same as the ones in your working directory. In our experience, Bismark duplicated a part of the filenames in the output files. We removed this duplicated portion. If you find the same behavior, you have to **adjust the filenames** of the output files or the names in the line of the R script that starts with file.list. The numbers of the differentially methylated cytosine positions are stored in two files, one named numbers25hyperDP and the other numbers25hypoDP.

The information stored in these two files can be used to identify the genomic fragments where the differentially methylated cytosine positions are situated. This is possible because, as described above, one of the output files of creepi (input_file_name_fragment_numbers.txt) contains the fragments and the start and end positions they have in the mock genome. The program **seek_fragments** uses the file with the positions of the differentially methylated cytosines (one position per line) and the creepi output to extract the fragments. Put the files with the positions of differentially methylated cytosines (numbers25hyperDP and numbers25hypoDP) and the file with genome fragments and their positions in the mock genome (catalog.tags_fragment_numbers txt) together with a copy of seek_fragments and the script_for_seek_fragments.sh in a directory. You can use the script directly or call seek fragments with the following options:

-p: file_with_differentially_methylated_cytosine_positions (e.g. numbers25hyperDP)

-f: file_with_fragments_and_position_numbers (e.g. catalog.tags_fragment_numbers.txt

-o: desired output_file_in_fasta_format

This output can then be used for a BLAST search or other applications you want to use them for.

To illustrate the whole process the Supplemental Figure 4 shows a summary in form of a flowchart with an overview of all the pipeline programs and important program parameters.

**Additional tool for calculating the bisulfite conversion rate**

In addition to the pipeline, we are also presenting a program to calculate the bisulfite conversion rate called conversion_calc). The program uses three input flags: -i for the input file (any fastq file); -o for the output file where the results are written (in addition to the screen); and –p for the position of the unmethylated cytosine in the adapter sequence. In our case, we need the reverse reads, as in the adapter part of the forward reads all cytosines are methylated after the nick translation step. If the P2 adapter sequence presented in Table 2 of the main text is used, the position of the unmethylated cytosine is 13. The program prints the input filename, the number of tymines and cytosines at the given position and the proportion of cytosines converted to tymines in percent (100 * (T/(T+C)).

**Supplementary Table 1.** Major cost factors associated with the cre-epiGBS protocol in comparison with previously published protocols for reference-free reduced representation bisulfite sequencing (epiGBS and BsRADseq). Materials like pipette tips, Eppendorf tubes, etc. are not taken into account. The DNA isolation kit is optional, and DNA isolation can be done by any protocol that assures high quality genomic DNA. Our protocol can use preexisting standard GBS adapters. In this case, only one hemimethylated P2 adapter needs to be newly synthesized. In the case of new P1 adapters, a combination of 32 unmethylated barcoded P1 adapters with three hemimethylated and barcoded P2 adapters is the most economical solution with the price we used for our calculations (€0.25 for each unmethylated base and €15 for each 5m-cytosine). In the case of a two-enzyme approach, we calculated with an 8 x 12 scheme due to its straightforward lab implementation using 96 well plates. Variable costs are given in bold face.

| **Cost factor** | **Cre-epiGBS 1 enzyme using preexisting GBS adapters** | **Cre-epiGBS 1 enzyme using new P1 adapters** | **Cre-epiGBS 2 enzymes** | **epiGBS†** | **BsRADseq‡** |
| --- | --- | --- | --- | --- | --- |
| DNA isolation kit | 274 | 274 | 274 | 274 | 274 |
| Qubit DNA Kit | 109 | 109 | 109 | 109 | 109 |
| Restriction enzymes§ | **85** | **85** | **170** | **85-170** | **85** |
| Adapters§§ | **195** | **1,282** | **2,564** | **4,605** | **6,838** |
| Ligase | 91 | 91 | 91 | 91 | 91 |
| Blunting kit | **-** | **-** | **-** | **-** | **116** |
| 5m-Cytosine dNTP Mix | **132** | **132** | **132** | **132** | **-** |
| DNA polymerase I | **58** | **58** | **58** | **58** | **-** |
| Methylation kit | 274 | 274 | 274 | 274 | 274 |
| Kapa HiFi Uracil+ kit | 283 | 283 | 283 | 283 | 283 |
| Size selection kit | 74 | 74 | 74 | 74 | 74 |
| PCR primer | **30** | **30** | **30** | **30** | **17** |
| Illumina PE Sequencing | **1,760** | **1,760** | **1,760** | **1,760** | **3,520** |
| **Total cost in Euros (€)** | **3,365** | **4,452** | **5,819** | **7,775 – 7,860** | **11,681** |

† epiGBS version with hemimethylated adapters. Prices for one or two enzymes are given.

‡ BsRADseq: Although not mentioned by the authors, it should be possible to drastically reduce the cost of adapters by using hemimethylated adapters without the nick translation step, because only the bottom strands survive due to the Y-shape of the P2 adapter. In this case, adapters should be around €4,600.

§ The cost of restriction enzymes depends on the choice of enzyme(s). We have used the price of PstI-HF (NEB) as a reference.

§§ The cost of adapters depends mainly on the charge for 5m-cytosine. We calculated with €15 for each position. Some major companies are much more expensive (€38–€40). Other factors include the sequences of barcodes and other technical regions that were estimated, but this depends on the decisions made by the users.

**Supplementary Figure 1.** Indications for de novo design of epiGBS adapters and PCR primers. **A**. Adapter sequences. The adapters are composed of a part of the Illumina specific sequences needed for bridge amplification and the sequencing process (lilac and black), three wobble bases (blue), a barcode sequence (red), and an enzyme specific sequence (green). The Illumina specific sequences will be extended during the PCR amplification. In order to be able to calculate the cytosine conversion rate, depending on the restriction enzyme recognition site, it may be necessary to include an unmethylated cytosine between the barcode sequence and the enzyme specific bases at the bottom strand of the P2 adapter. The lilac part of the adapters is identical to the 3´portion of the PCR primers. The P1 and P2 adapters contain three wobble positions each. The wobbles of P2 are restricted to H (= A, C or T; upper strand) and D (= A, G or T; lower strand) because the bisulfite treatment would convert the cytosines of the lower strand to Uracil. The barcodes of the P1 adapter should be of variable length in order to avoid phasing problems. This is not necessary in the case of the P2 adapter, where a uniform barcode length of four bases can be used. For barcode design it is advisable to use specific software due to the various conditions barcodes should fulfill. **B**. Three examples for the enzyme specific sequences of the adapters. Two of the example enzymes have 5´produding ends (ApeKI and HpaII), one 3´produding ends (PstI). The adapters are designed with the aim that after ligation of the genomic DNA fragment to the adapters, the recognition site of the restriction enzyme is not reconstituted. Some computer programs used to design barcodes integrate this position, as we indicate in the case of ApeKI. For PstI and HpaII we show a design where the first enzyme specific base of the P1 adapter is fixed. In the case of ApeKI and PstI it is possible to integrate an unmethylated cytosine in the enzyme specific part of the bottom strand of the P2 adapter. This is not possible in the case of HpaII, because an unmethylated cytosine in the only enzyme specific position of the bottom strand of the P2 adapter would reconstitute the cut site. Therefore, an unmethylated cytosine is placed between barcode and the enzyme specific part of the P2 adapter. **C**. Primer sequences that can be used with the given P1 and P2 adapter sequences. The PCR-F primer is shorter than the PCR primer presented in the main text. This is possible because the P1 adapters we used were significantly shorter than the adapter sequences suggested here.

**Supplementary Figure 2.** Changes of the original recognition sites along the progress of the lab protocol. The adapter sequences are designed to avoid the reconstitution of the recognition sequence of the restriction enzymes. The bioinformatics pipeline trims this changed base (in pink). At the end of the process, the bases marked in red remain. TACAA and CTACA are the sequences we expect at end of the joined sequence reads in the case of PstI sites. The parameters to use are therefore -1 CTACA and -5 TACAA. For ApeKI, we expect CAAC, CTAC, ACAA and ACTA. Parameters to pass to creepi: -1 ACTA; -2 ACAA; -5 CAAC; -6 CTAC. Rust color is used to highlight bases used to calculate the cytosine conversion rate.

**Supplementary Figure 3.** How to find the sequence parameters of creepi in the case of a two-restriction enzyme combination. The example of PstI – ApeKI is shown. **A**. Genomic fragments prior to the restriction cut. **B**. Genomic fragments after restriction ligation. **C**. Genomic fragments after bisulfite treatment. **D**. After PCR: the original top chain does not amplify. Reads (top chain) correspond to the complement of the bottom chain. To find the final parameter values for creepi use only the four innermost bases of the PstI recognition sites (shown in red). Parameters to pass to creepi: -1 ACTG; -2 ACAA; -3 CTAC; -5 ACAA; -6 CAAC; -7 CTAC. Pink color indicates bases introduced in the adapter sequences to avoid the reconstitution of the restriction enzyme cut site, rust color is used to highlight bases used to calculate the cytosine conversion rate.


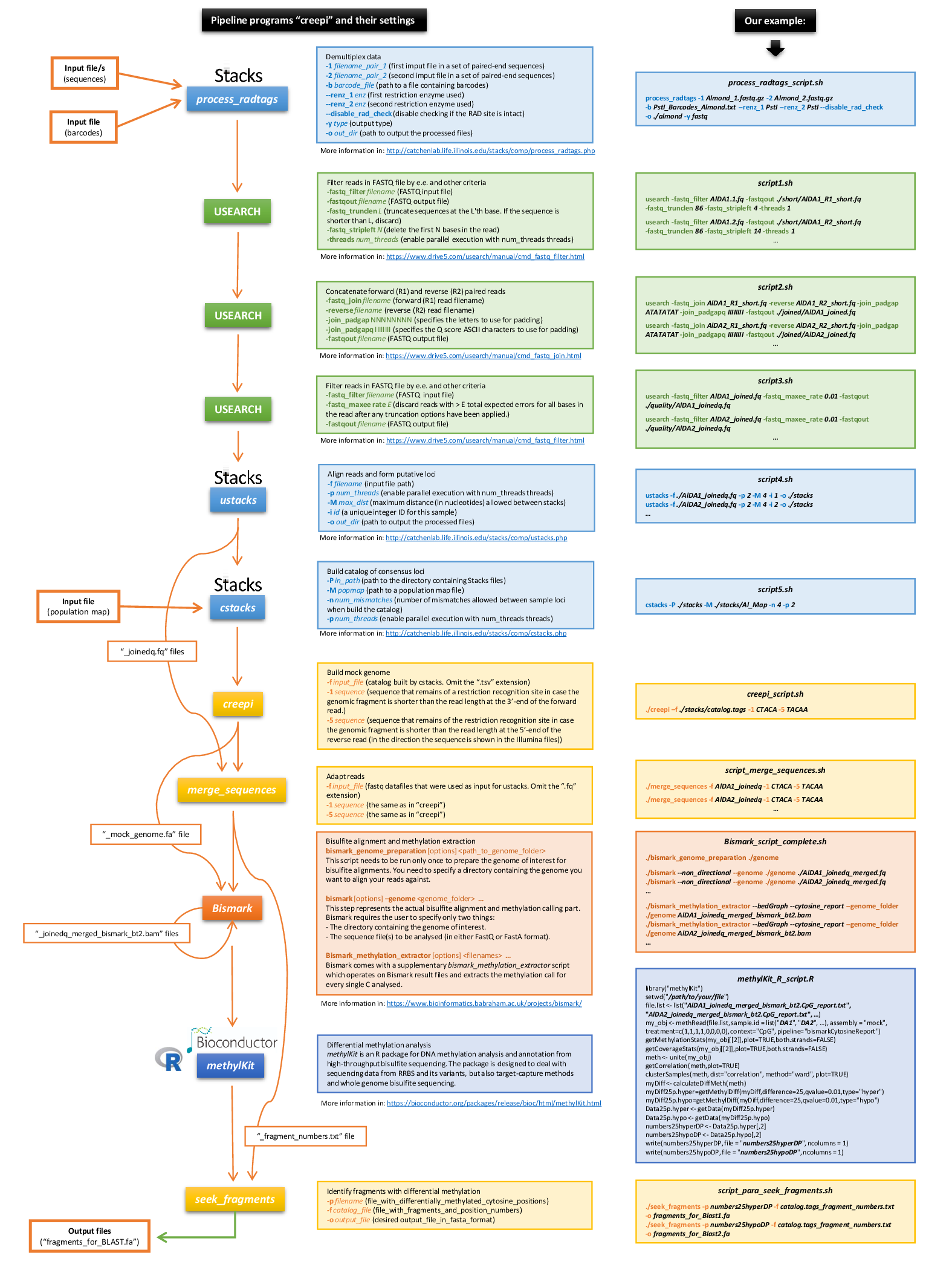


**Supplementary Figure 4.** Flowchart showing the pipeline programs and the example scripts used in this supplement to obtain information on differentially methylated cytosines.
